# Rates and determinants of early initiation of breastfeeding and exclusive breast feeding at 42 days postnatal in six low and middle-income countries: A prospective cohort study

**DOI:** 10.1186/1742-4755-12-S2-S10

**Published:** 2015-06-08

**Authors:** Archana Patel, Sherri Bucher, Yamini Pusdekar, Fabian Esamai, Nancy F Krebs, Shivaprasad S Goudar, Elwyn Chomba, Ana Garces, Omrana Pasha, Sarah Saleem, Bhalachandra S Kodkany, Edward A Liechty, Bhala Kodkany, Richard J Derman, Waldemar A Carlo, K Michael Hambidge, Robert L Goldenberg, Fernando Althabe12, Mabel Berrueta, Janet L Moore, Elizabeth M McClure, Marion Koso-Thomas, Patricia L Hibberd

**Affiliations:** 1 Indira Gandhi Government Medical College and Lata Medical Research Foundation, Nagpur, India; 2 Indiana University School of Medicine, Indianapolis, IN, USA; 3 Moi University, Eldoret, Kenya; 4 University of Colorado School of Medicine, Denver, CO, USA; 5 KLE University’s Jawaharlal Nehru Medical College, Belgaum, India; 6 University Teaching Hospital, Lusaka, Zambia; 7 FANCAP, Guatemala City, Guatemala; 8 Aga Khan University, Karachi, Pakistan; 9 Christiana Health Care Services, Newark, DE, USA; 10 University of Alabama at Birmingham, Birmingham, AL, USA; 11 Columbia University, New York, NY, USA; 12 IECS, Buenos Aires, Argentina; 13 RTI International, Durham, NC, USA; 14 Eunice Kennedy Shriver National Institute of Child Health and Human Development of the US National Institutes of Health, Bethesda, MD, USA; 15 Massachusetts General Hospital for Children, Boston, USA

**Keywords:** Early initiation of breastfeeding, exclusive breastfeeding, neonatal mortality, global health, newborn

## Abstract

**Background:**

Early initiation of breastfeeding after birth and exclusive breastfeeding through six months of age confers many health benefits for infants; both are crucial high impact, low-cost interventions. However, determining accurate global rates of these crucial activities has been challenging. We use population-based data to describe: (1) rates of early initiation of breastfeeding (defined as within 1 hour of birth) and of exclusive breastfeeding at 42 days post-partum; and (2) factors associated with failure to initiate early breastfeeding and exclusive breastfeeding at 42 days post-partum.

**Methods:**

Prospectively collected data from women and their live-born infants enrolled in the Global Network’s Maternal and Newborn Health Registry between January 1, 2010-December 31, 2013 included women-infant dyads in 106 geographic areas (clusters) at 7 research sites in 6 countries (Kenya, Zambia, India [2 sites], Pakistan, Argentina and Guatemala). Rates and risk factors for failure to initiate early breastfeeding were investigated for the entire cohort and rates and risk factors for failure to maintain exclusive breastfeeding was assessed in a sub-sample studied at 42 days post-partum.

**Result:**

A total of 255,495 live-born women-infant dyads were included in the study. Rates and determinants for the exclusive breastfeeding sub-study at 42 days post-partum were assessed from among a sub-sample of 105,563 subjects. Although there was heterogeneity by site, and early initiation of breastfeeding after delivery was high, the Pakistan site had the lowest rates of early initiation of breastfeeding. The Pakistan site also had the highest rate of lack of exclusive breastfeeding at 42 days post-partum. Across all regions, factors associated with failure to initiate early breastfeeding included nulliparity, caesarean section, low birth weight, resuscitation with bag and mask, and failure to place baby on the mother’s chest after delivery. Factors associated with failure to achieve exclusive breastfeeding at 42 days varied across the sites. The only factor significant in all sites was multiple gestation.

**Conclusions:**

In this large, prospective, population-based, observational study, rates of both early initiation of breastfeeding and exclusive breastfeeding at 42 days post-partum were high, except in Pakistan. Factors associated with these key breastfeeding indicators should assist with more effective strategies to scale-up these crucial public health interventions.

**Trial registration:**

Registration at the Clinicaltrials.gov website (ID# NCT01073475).

## Background

Breast milk, recommended as the best feeding option for neonates and young infants, provides many immunological, psychological, social, economic, and environmental benefits. The global recommendations of the World Health Organization (WHO) are that (1) all infants should start breastfeeding within one hour of birth (early initiation of breastfeeding, EIBF) and (2) be exclusively breastfed (EBF; only breast milk, no other liquids or solids, not even water, with the exception of oral rehydration solution [ORS], or drops/syrups of vitamins, minerals or medicines) up to 6 months of age, then partially breastfed thereafter as part of a comprehensive complementary feeding strategy up to 2 years of age [1]. EIBF and EBF are also recommended for HIV-infected women who receive combination antiretroviral treatment regimens for prevention of mother-to-child transmission of HIV; early cessation of breastfeeding has been associated with a significantly increased risk of morbidity among older, HIV-exposed African children [2].

EIBF is low-cost and has substantial potential to reduce neonatal and early infant morbidity [3-7] and mortality [8-10]. Despite these benefits, less than 40% of infants in resource limited settings are breastfed within an hour of birth [11]. Similarly, despite the recommendation for EBF up to age six months, global rates of EBF at six months of age are low [12] and EBF rates fall within a few weeks after birth [13,14]. Identifying barriers and facilitators to EIBF and EBF is important in order to develop feasible and sustainable strategies by which to improve global coverage of these key public health interventions.

Much of the data on rates and determinants of EIBF and EBF come from national Demographic and Health Surveys (DHS) [15-18]. These cross-sectional surveys are retrospective and rely on mother’s recall of timing of initiation of breast feeding and duration of EBF; as such, they may not provide accurate information on either the population-based rates of, or barriers to, EIBF and EBF. To address these methodological limitations and gaps in the current global evidence-base, we conducted a secondary analysis of data prospectively collected in the multi-country, population based Maternal and Newborn Health Registry (MNHR) of the Global Network for Women’s and Children’s Health Research (Global Network) [19]. The objectives of the study were to prospectively investigate in low-resource global regions: (1) overall, regional, and site-specific rates of EIBF prior to one hour after delivery and EBF at 42 days post-partum; and (2) factors associated with failure to achieve EIBF and EBF.

## Methods

### Study design and setting

The study was conducted using prospectively collected data from 106 clusters at 7 sites in six countries participating in the MNHR, conducted between January 1, 2010 and December 31, 2013. The MNHR is supported by the *Eunice Kennedy Shriver* National Institute of Child Health and Human Development’s (NICHD’s) Global Network, a multi-site research network representing partnerships of U.S. and international investigators at study sites in Argentina, Guatemala, India (2 sites; Nagpur and Belgaum), Pakistan, Kenya, and Zambia. Detailed methods utilized by the MNHR have been previously published [19].

### Participants

Briefly, pregnant women are registered either at the earliest point of contact with the public health system or via active surveillance in the study communities (“clusters”) by MNHR public health staff. The women are followed throughout pregnancy, after delivery at a perinatal follow-up visit, and through 42 days after birth to obtain a variety of maternal and infant outcomes. Study data are collected by trained registry administrators, generally nurses or health workers, with oversight by local and central investigators.

### Ethics review

The Institutional Review Boards and Ethics Research Committees of the participating institutions, and the Ministries of Health of the respective countries approved the MNHR. Prior to initiation of the study, approval was obtained from the participating communities through sensitization meetings. Individual informed consent for study participation is requested from each study participant. No monetary reimbursements are provided to study participants nor to the communities participating in the study. A Data Monitoring Committee, appointed by the NICHD, oversees and reviews the study at annual meetings.

### Data collection procedures

Data in the MNHR include socio-demographic variables, obstetric history, and health care seeking behavior during the antenatal and postnatal periods, delivery outcomes, maternal and newborn complications, recommendations received about breastfeeding, and referrals and health status of the mother/infants. In addition to enrolment during pregnancy, two postnatal visits were conducted: the first within one week after delivery (perinatal visit) and the other at day 42 post-partum. All study data were obtained by trained interviewers who were unaware of the study hypotheses and recorded maternal responses on standardized case report forms. Data were collected and entered and edited at each study site and transmitted through secure methods to a central data coordinating center (RTI International). All analyses were performed with SAS version 9.3 (SAS Institute, Cary, NC, USA).

## Statistical analyses

### Outcomes

EIBF was defined as initiation of breastfeeding within one hour after delivery, based on the maternal report, at the perinatal visit, of how soon after birth the child was given breast milk. Exclusive breastfeeding at 42 days of life was defined as the baby having received no other food, liquids, or substances (exclusive of medication, immunizations, ORS drops, or vitamin supplements) other than breast milk at the 6-week follow-up visit.

### Analysis

We calculated the overall rates of EIBF and EBF at 42 days of life and also examined rates by global region and GN site. Because of cultural differences associated with the sites, we examined these factors by region. To assess regional differences, we grouped the two sub-Saharan African sites (Zambia and Kenya), the two Indian sites (Nagpur and Belgaum, India), and Latin American sites (Argentina and Guatemala) and considered the Pakistan site separately. The models were developed for both EIBF and EBF with covariates examined for each region or site separately.

Based on demographic factors associated with breastfeeding in the literature, we first evaluated the individual association of factors with breastfeeding using a cut-off of p < 0.1 for each region. Then using the demographic characteristics significant in at least one region as covariates, we developed a multivariable model to assess the risk of EIBF and EBF associated with each. As a final step, we developed a reduced model with only the factors that remained significant in the multivariate multivariable model and calculated point and interval estimates of risk ratios using multivariable generalized linear regression models with a Poisson distributional assumption and a log link. We used the empirical covariance matrix with generalized estimating equations to account for correlation of outcomes within clusters to assure appropriately sized p-values and confidence intervals.

For a sub-sample of the study participants, we also explored the association of the co-variables (including lack of EIBF) on not exclusively breastfeeding by day 42 for infants alive at day 42. Because many very low birthweight infants (<1500 g) were not alive at 42 days, we utilized two birth weight categories (<2500 grams, >= 2500 grams), rather than multiple birth weight categories as in the EIBF model. Additionally, the Argentina site did not participate in this sub-study. All other aspects of the modelling for the EBF analysis were the same as for EIBF.

## Results

### Early initiation of breastfeeding

#### Enrollment Flow Diagram (Figure 1)

**Figure 1:**
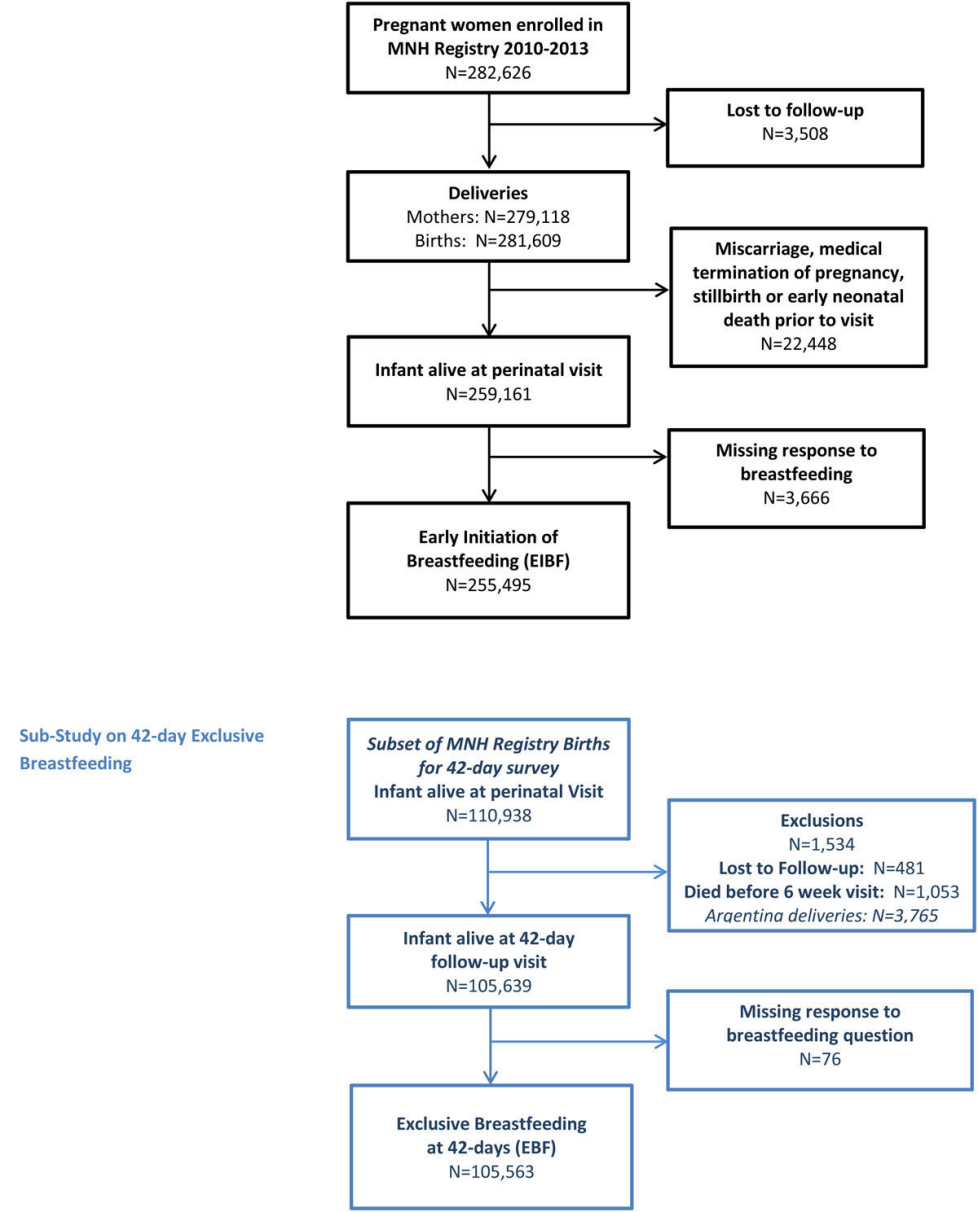
CONSORT Diagram for study and exclusive breastfeeding at 42 day sub-study

**Figure 2:**
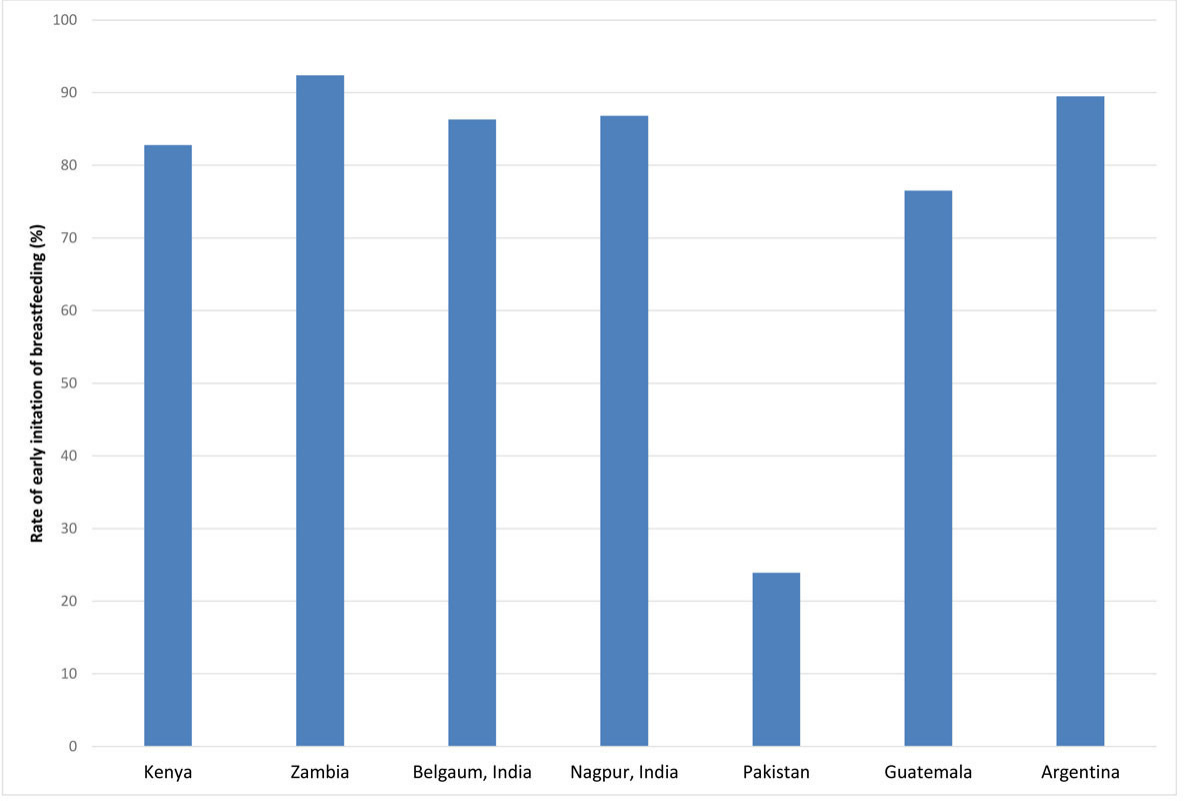
Rates of early initiation of breastfeeding within one hour of delivery in the Global Network sites by region, 2010-2013

During the study period, 282,626 women were enrolled in the MNHR of which 3,508 were lost to follow-up. There were 259,161 live infants at the perinatal follow-up visit. Our sample included 255,495 women who had responded to the question about whether EIBF had occurred. This included 61,232 women from the African sites (24% of total sample), 157,834 women from the Asian sites (61% of total sample) and 38,159 women from the Latin American sites (15% of total sample). Figure 2 shows the rates of EIBF at the different sites, ranging from 23.9% in the Pakistan site to 92.4% in the Zambian site.

#### Demographic characteristics

Overall, 84% of the mothers were 20 to 35 years of age (Table 1). About 24% of the population overall had no formal education; however, in the Pakistan site, 83% of women lacked formal education. Women with primary education comprised 63% in the sites in Africa and Latin America whereas in the Indian sites, 25% had primary education and 48% had secondary education. Parity greater than two was observed in 47% of women in the Pakistan site, about one-third of women in the African and Latin American sites, and only in 5% of women from the sites in India. Having an initial antenatal visit in the first trimester ranged from 3.8% in Kenya to more than 60% in both Indian sites. Rates of caesarean section were 12.4% overall and highest in the Argentina site while <2% of women in the African sites reported delivery by caesarean section. Only 2% of women were delivered by physicians in Africa, with most women reporting nurse/midwives, traditional birth attendants (TBAs) or family members as birth attendants. At the Indian sites, 96% of the deliveries were conducted by physicians or nurse/midwives, as compared to 52% in the Pakistan and 72% in the Latin American sites. The remaining deliveries in the Pakistan and Latin American sites were conducted by TBAs. The rates of multiple births (1.5%) and gender ratios were similar across study sites. The low birth weight rate (<2500 g) was about 10% overall, with highest rates in the Indian and Pakistan sites. Women in the Pakistan site also reported the highest rates of newborn resuscitation, at 4.6%, and had very low rates of the baby being placed on the mother’s chest (6.6%).

**Table 1:** Demographic characteristics of women in the Global Network’s Maternal Neonatal Health Registry by site in the years 2010 - 2013

	African Sites		Indian Sites			Latin America Sites		
	Kenya	Zambia	Belgaum	Nagpur	Pakistan	Argentina	Guatemala	Total

Maternal age, N (%)								

< 20	7,503 (21.7)	6,712 (25.2)	7,250 (9.5)	725 (1.9)	1,694 (3.9)	2,636 (27.3)	4,663 (16.4)	31,183 (12.1)

20-35	25,575 (74.1)	17,832 (67.0)	68,858 (90.3)	36,634 (97.8)	39,804 (90.7)	6,293 (65.1)	20,837 (73.3)	215,833 (84.0)

> 35	1,421 (4.1)	2,086 (7.8)	124 (0.2)	97 (0.3)	2,389 (5.4)	738 (7.6)	2,942 (10.3)	9,797 (3.8)

Education, N (%)								

No formal education	1,048 (3.0)	2,789 (10.5)	15,611 (20.6)	1,140 (3.0)	36,256 (82.7)	243 (2.5)	5,454 (19.2)	62,541 (24.4)

Primary	24,636 (71.4)	14,573 (55.0)	25,060 (33.1)	6,440 (17.2)	3,398 (7.7)	5,993 (62.5)	17,943 (63.1)	98,043 (38.3)

Secondary	7,581 (22.0)	8,669 (32.7)	28,003 (37.0)	22,260 (59.5)	2,648 (6.0)	3,201 (33.4)	4,759 (16.7)	77,121 (30.1)

University+	1,243 (3.6)	473 (1.8)	7,045 (9.3)	7,596 (20.3)	1,554 (3.5)	149 (1.6)	288 (1.0)	18,348 (7.2)

Parity, N (%)								

0	8,609 (24.9)	7,170 (26.9)	32,203 (42.5)	17,953 (47.9)	9,041 (20.6)	3,165 (32.9)	7,950 (27.9)	86,091 (33.6)

1-2	13,403 (38.8)	10,037 (37.7)	38,592 (50.9)	18,553 (49.5)	14,259 (32.5)	3,746 (38.9)	10,259 (36.1)	108,849 (42.4)

> 2	12,496 (36.2)	9,438 (35.4)	5,041 (6.6)	970 (2.6)	20,624 (47.0)	2,723 (28.3)	10,242 (36.0)	61,534 (24.0)

Trimester for first ANC visit, N (%)								

First	1,232 (3.8)	2,169 (8.3)	46,710 (63.1)	28,973 (77.5)	8,575 (24.1)	3,213 (37.3)	11,429 (41.5)	102,301 (42.3)

Second	19,101 (58.8)	18,823 (71.9)	23,144 (31.2)	7,317 (19.6)	11,425 (32.1)	3,873 (45.0)	12,146 (44.1)	95,829 (39.6)

Third	12,133 (37.4)	5,182 (19.8)	4,209 (5.7)	1,075 (2.9)	15,606 (43.8)	1,525 (17.7)	3,994 (14.5)	43,724 (18.1)

Delivery mode, N (%)								

Vaginal	33,666 (97.4)	26,173 (98.1)	65,197 (85.4)	29,568 (78.9)	37,468 (85.1)	6,294 (64.9)	23,118 (81.2)	221,484 (86.1)

Vaginal assisted	410 (1.2)	235 (0.9)	208 (0.3)	417 (1.1)	2,428 (5.5)	13 (0.1)	24 (0.1)	3,735 (1.5)

C-section	477 (1.4)	271 (1.0)	10,902 (14.3)	7,498 (20.0)	4,144 (9.4)	3,394 (35.0)	5,313 (18.7)	31,999 (12.4)

Birth attendant, N (%)								

Physician	596 (1.7)	546 (2.0)	44,515 (58.3)	22,495 (60.0)	11,140 (25.3)	7,011 (72.3)	12,246 (43.0)	98,549 (38.3)

Nurse/Midwife/HW	14,002 (40.5)	14,916 (55.9)	27,615 (36.2)	13,736 (36.6)	11,691 (26.5)	2,641 (27.2)	511 (1.8)	85,112 (33.1)

TBA	15,749 (45.6)	6,954 (26.1)	1,867 (2.4)	1,015 (2.7)	20,400 (46.3)	2 (0.0)	15,616 (54.9)	61,603 (24.0)

Family/Other	4,206 (12.2)	4,263 (16.0)	2,310 (3.0)	237 (0.6)	810 (1.8)	42 (0.4)	83 (0.3)	11,951 (4.6)

Live births (neonates), N	34,931	26,875	76,782	37,714	44,472	9,763	28,624	259,161

Multiple birth, N (%)	740 (2.1)	398 (1.5)	940 (1.2)	474 (1.3)	878 (2.0)	124 (1.3)	345 (1.2)	3,899 (1.5)

Male gender, N (%)	17,653 (50.5)	14,126 (52.6)	39,775 (51.8)	19,612 (52.0)	23,220 (52.2)	5,040 (51.7)	14,549 (50.8)	133,975 (51.7)

Birth weight	34,902	26,871	76,770	37,700	44,413	9,756	28,621	259,033

< 1000g	3 (0.0)	11 (0.0)	33 (0.0)	5 (0.0)	14 (0.0)	16 (0.2)	16 (0.1)	98 (0.0)

1000-1499g	24 (0.1)	42 (0.2)	289 (0.4)	152 (0.4)	209 (0.5)	34 (0.3)	81 (0.3)	831 (0.3)

1500-2499g	836 (2.4)	1,151 (4.3)	9,396 (12.2)	5,128 (13.6)	5,990 (13.5)	483 (5.0)	3,355 (11.7)	26,339 (10.2)

≥ 2500g	34,039 (97.5)	25,667 (95.5)	67,052 (87.3)	32,415 (86.0)	38,200 (86.0)	9,223 (94.5)	25,169 (87.9)	231,765 (89.5)

Bag and mask resuscitation, N (%)	531 (1.5)	460 (1.7)	2,688 (3.5)	1,034 (2.8)	2,027 (4.6)	369 (3.8)	285 (1.0)	7,394 (2.9)

Baby placed on mother's chest after delivery, N (%)	20,231 (58.2)	20,853 (78.6)	43,337 (58.6)	23,066 (62.5)	2,920 (6.6)	7,563 (78.5)	14,602 (51.4)	132,572 (52.1)

#### Factors associated with lack of EIBF (Table 2)

In the adjusted, multivariable model, the common statistically significant determinants of lack of EIBF across the regions were nulliparity, caesarean section, low birth weight, resuscitation with bag and mask and failure to place baby on the mother’s chest after delivery. In the African sites, older maternal age was also associated with lack of EIBF. Across the sites, lower levels of maternal education were associated with a slight increase in lack of EIBF but results were not consistent. In the Pakistan site, EIBF was more likely if the delivery was conducted by a TBA. This association was also observed in the Latin American sites. Male babies were significantly less likely to receive EIBF in the African and Latin American sites.

**Table 2:** Factors associated with lack of early initiation of breastfeeding within Global Network sites by region for the years 2010 -2013*

	African Sites		Indian Sites		The Pakistan Site		Latin American Sites	
	**%**	**RR (95% CI), P value**	**%**	**RR (95% CI), P value**	**%**	**RR (95% CI), P value**	**%**	**RR (95% CI), P value**

Maternal Age		0.0052		NS		NS		NS

< 20	23.4	1.02 (0.97, 1.08), 0.4577	5.7		3.9		21.8	

20-35	70.6	1.0	94.0		90.7		69.2	

> 35	6. 0	1.09 (1.03, 1.15), 0.0014	0.3		5.4		9.0	

Education		<.0001		0.0003		0.0121		NS

No formal education	6.8	1.19 (0.99, 1.43), 0.0668	11.8	0.95 (0.76, 1.17), 0.6094	82.7	1.05 (1.01, 1.08), 0.0057	10.8	

Primary	63.2	1.16 (1.03, 1.30), 0.0124	25.1	1.24 (0.92, 1.67), 0.1511	7.8	1.04 (1.0, 1.07),0.0250	62.9	

Secondary	27.3	1.06 (0.94, 1.20), 0.3487	48.3	1.03 (0.95, 1.12), 0.4557	6.0	1.0 (0.97, 1.04),0.7766	25.0	

University or higher	2.7	1.0	14.8	1.0	3.5	1.0	1.3	

Parity		0.0013		0.0236		<.0017		<.0001

0	25.9	1.15 (1.01, 1.31), 0.0294	45.2	1.04 (1.0, 1.08), 0.0353	20.5	1.02 (1.01, 1.04), <.0006	30.4	1.19 (1.09, 1.29), <.0001

1-2	38.3	1.0	50.2	1.0	32.5	1.0	37.5	1.0

> 2	35.8	0.94 (0.91, 0.98), 0.0013	4.6	1.16 (1.0, 1.35), 0.0489	47.0	1.02 (1.00, 1.03), <.0610	32.1	0.94 (0.90, 0.99), <.0245

Trimester of first ANC		NS		0.0279		NS		NS

First	6.1		70.3	1.0	24.1		39.4	

Second	65.3		25.4	0.76 (0.58, 0.99), 0.0420	32.1		44.5	

Third	28.6		4.3	0.49 (0.29, 0.84), 0.0093	43.8		16.1	

Delivery mode		<.0001		<.0001		<.0001		<.0001

Vaginal	97.8	1.0	82.2	1.0	85.1	1.0	73.0	1.0

Vaginal assisted	1.0	1.26 (0.97, 1.62), 0.0796	0.7	0.75 (0.33, 1.71), 0.4892	5.5	1.07 (1.03, 1.11), 0.0002	0.1	1.24 (0.71, 2.17), 0.4480

C-section	1.2	2.06 (1.67, 2.54), <.0001	17.1	3.76 (1.77, 7.99), 0.0006	9.4	1.21 (1.13, 1.29), <.0001	26.9	2.26 (1.74, 2.93), <.0001

Birth attendant		NS		0.0001		0.0020		<.0001

Physician	1.9		59.1	1.0	25.3	1.0	57.6	1.0

Nurse/Midwife/HW	48.2		36.5	0.75 (0.55, 1.04), 0.0819	26.6	1.02 (0.97, 1.06), 0.4955	14.6	0.95 (0.69, 1.30), 0.7309

TBA	35.8		2.6	0.87 (0.43, 1.78), 0.7077	46.3	0.95 (0.91, 1.00), 0.0410	27.5	0.56 (0.41, 0.77), <.0004

Family/Other	14.1		1.8	2.22 (1.04, 4.71), 0.0385	1.8	1.0 (0.94, 1.06), 0.9867	0.3	1.37 (1.14, 1.64), 0.0008

Multiple birth		<.0001		NS		<.0001		NS

Yes	1.8	1.65 (1.47, 1.86), <.0001	1.2		2.0	1.08 (1.04, 1.11), <.0001	1.2	

No	98.2	1.0	98.8		98.0	1.0	98.8	

Gender		0.0077						<0.0008

Male	51.6	1.04 (1.01, 1.07), 0.0077	51.9	NS	52.2	NS	51.2	1.07 (1.03, 1.12), <.0008

Female	48.4	1.0	48.1		47.8		48.8	1.0

Birth weight		<.0001		<.0001		<.0001		<.0001

< 1000g	0.0	3.07 (2.09, 4.51), <.0001	0.0	3.32 (1.57, 6.99), 0.0016	0.0	1.29 (1.17, 1.41), <.0001	0.1	2.03 (1.60, 2.57), <.0001

1000-1499g	0.1	2.42 (1.94, 3.02), <.0001	0.4	2.63 (1.83, 3.77), <.0001	0.5	1.08 (0.99, 1.18), 0.0707	0.3	2.25 (1.90, 2.66), <.0001

1500-2499g	3.4	1.48 (1.32, 1.65), <.0001	12.9	1.33 (1.06, 1.68), 0.0134	13.5	1.05 (1.03, 1.08), <.0001	8.4	1.35 (1.23, 1.47), <.0001

≥ 2500g	96.5	1.0	86.7	1.0	86.0	1.0	91.2	1.0

Bag and mask resuscitation		<.0001		<.0001		<.0001		<.0001

Yes	1.6	2.15 (1.78, 2.61), <.0001	3.1	2.22 (1.84, 2.69), <.0001	4.6	1.10 (1.05, 1.15), <.0001	2.4	2.05 (1.74, 2.41), <.0001

No	98.4	1.0	96.9	1.0	95.4	1.0	97.6	1.0

Baby on mother's chest after delivery		<.0001		<0.0001		<.0001		<.0001

Yes	68.4	1.0	60.6	1.0	6.6	1.0	64.9	1.0

No	31.6	1.98 (1.65, 2.37), <.0001	39.4	4.34 (2.67, 7.05), <.0001	93.4	1.67 (1.46, 1.91), <.0001	35.1	3.30 (2.32, 4.68), <.0001

*Poisson multivariable reduced model with generalized estimating equations accounting for cluster; NS = Not significant

### Exclusive breastfeeding on day 42 of life

For a sample of the original cohort, a survey was conducted at the 42-day follow-up visit to assess factors associated with EBF. This survey was conducted for mothers who had received ANC during pregnancy whose infants were alive at 42 days postnatal. The Argentinian site did not participate. The Kenyan and Zambian sites contributed 23,056 deliveries (22%), the Asian sites contributed 66,118 deliveries (63%) and the Guatemalan site 15,597 (15%). EIBF in this subsample was slightly lower (63%) than for the entire study period (75% as reported above), but otherwise the demographic characteristics of women were similar to that of the main cohort (data not shown).

#### Rates of exclusive breastfeeding on day 42 of life (Figure 3)

**Figure 3:**
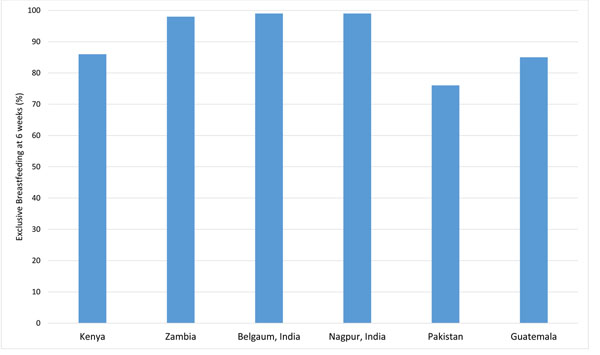
Rates of exclusive breastfeeding at 6-weeks in the Global Network sites by region, 2010-2013

Rates of EBF at 42 days after birth ranged from 76% to 99.5% across participating Global Network sites. The Indian sites reported the highest rates of EBF at the 42-day follow-up visit (Belgaum, 99.5%; Nagpur, 99.0%), followed by the sites in Zambia (98.7%), Kenya (85.5%), and Guatemala (84.6%). The lowest rate of EBF at 42 days after birth was observed in the Pakistan site (75.9%).

#### Factors associated with lack of exclusive breastfeeding on day 42 of life (Table 3)

In the adjusted, multivariable model, multiple birth was a significant risk factor for failure to EBF across all sites. In the African sites, 15% of women did not EBF and the associated risk factors were lower (<20) or higher (>35) maternal age. In the Guatemalan site, 15% of women also reported failure to EBF. Factors associated with lack of EBF in this setting included: maternal age >35 years, nulliparity, LBW, resuscitation of the newborn, and lack of EIBF. Among women in the Guatemalan site, younger maternal age, lower education levels, delivery by TBAs and late initiation of ANC were associated with higher rates of EBF at 42 days postnatal. In the Pakistan site, factors that were associated with failure to achieve EBF also included maternal age >35 years, delivery by caesarean section, and nulliparity. Factors that were not significantly associated with EBF included infant gender, delivery mode and placement of infant on mother’s chest after birth (data not shown).

**Table 3:** Factors associated with lack of exclusive breast feeding within Global Network sites by region for the years 2010 -2013*

	African Sites		Indian Sites		The Pakistan Site		The Guatemalan Site	
	**%**	**RR (95% CI), P value**	**%**	**RR (95% CI), P value**	**%**	**RR (95% CI), P value**	**%**	**RR (95% CI), P value**

Maternal Age		0.0025		NS		0.0086		<.0001

< 20	24.3	1.15 (1.01, 1.31), 0.0390	5.1		3.9	0.90 (0.76, 1.06), 0.2009	16.1	0.81 (0.75, 0.89), <.0001

20-35	70.0	1.0	94.6		90.2	1.0	73.6	1.0

> 35	5.7	1.15 (1.01, 1.31), 0.0290	0.3		5.9	1.15 (1.05, 1.26), 0.0038	10.3	1.45 (1.30, 1.62),<.0001

Maternal education		<.0001		NS		NS		<.0001

No formal education	6.2	1.81 (1.05, 3.12), 0.0333	11.3		80.7		16.0	0.37(0.29, 0.48), <.0001

Primary	60.9	1.41 (0.86, 2.31), 0.1745	24.5		8.1		63.6	0.37(0.29, 0.48), <.0001

Secondary	29.6	1.18 (0.73, 1.90), 0.4939	48.1		7.0		19.2	0.60 (0.51, 0.70), <.0001

University or higher	3.3	1.0	16.1		4.2		1.3	1.0

Parity		0.0055		NS		0.0070		<.0001

0	27.2	0.85 (0.66, 1.10), 0.2220	43.9		20.2	1.10 (1.03, 1.16), 0.0019	28.6	1.20 (1.14, 1.26), <.0001

1-2	38.7	1.0	51.7		31.9	1.0	37.1	1.0

> 2	34.1	1.22 (1.07, 1.39), 0.0032	4.4		47.9	1.04 (0.97, 1.12), 0.2469	34.4	1.06 (0.97, 1.15), 0.1942

Trimester of first ANC		NS		0.0654		0.0699		0.0090

First	8.6		82.6	1.0	32.4	1.0	45.2	1.0

Second	59.7		14.9	1.33 (1.04, 1.70), 0.0213	27.7	0.93 (0.87, 1.00), 0.0629	41.3	0.94 (0.89, 0.99), 0.0127

Third	31.7		2.5	0.73 (0.34, 1.56), 0.4211	39.9	0.99 (0.92, 1.07), 0.8923	13.5	0.88 (0.79, 0.98), 0.0227

Birth attendant		0.0350		0.0027		NS		<.0001

Physician	2.0	1.0	62.4	1.0	30.0		51.3	1.0

Nurse/Midwife/HW	55.8	1.21 (0.84, 1.72), 0.3056	35.1	1.06 (0.87, 1.29), 0.5487	26.6		1.7	0.96 (0.71, 1.30), 0.7892

TBA	28.7	1.41 (0.92, 2.16), 0.1143	0.8	2.60 (1.47, 4.60), 0.0010	41.3		46.7	0.69 (0.64, 0.74), <.0001

Family/Other	13.5	1.43 (0.97, 2.09), 0.0674	1.7	0.67 (0.37, 1.20),0.1794	2.0		0.4	1.12 (0.67, 1.89) 0.6651

Multiple birth		<.0001		<.0001		<.0001		<.0001

Yes	1.8	2.27 (1.61, 3.19), <.0001	1.3	3.60 (2.17, 5.97), <.0001	2.0	1.99 (1.70, 2.34), <.0001	1.2	3.25 (2.75, 3.84), <.0001

No	98.2	1.0	98.7	1.0	98.0	1.0	98.8	1.0

Birth weight		NS		0.0378		NS		<.0001

< 2500 g	3.7		14.8	1.29 (1.01, 1.64), 0.0378	16.0		12.2	1.19 (1.11, 1.28), <.0001

≥ 2500 g	96.3		85.2	1.0	84.0		87.8	1.0

Bag and mask resuscitation		NS		NS		0.0657		0.0233

Yes	1.4		3.0		6.8	0.89 (0.79, 1.01), 0.0657	1.2	1.09 (1.01, 1.18), 0.0233

No	98.6		97.0		93.2	1.0	98.8	1.0

Timely initiation of breastfeeding		NS		0.0001		NS		0.0030

Yes	88.1		84.0	1.0	18.4		72.6	1.0

No	11.9		16.0	1.60 (1.25, 2.04 ), 0.0001	81.6		27.4	1.22 (1.07, 1.40), 0.0030

* Poisson multivariable reduced model with generalized estimating equations accounting for cluster; NS = Not significant

## Discussion

A major finding of our study was that the overall rate of EIBF was higher, at 75%, than has been typically reported in prior studies using DHS survey data [15-18,20-22]. We observed some site-specific variations in EIBF, with the lowest rate observed in Pakistan. Some variation in rates between sites may have been due in part to health system-wide disruptions in service delivery (e.g., floods in Pakistan, 2010; health worker strikes in Kenya, 2012). However, the lower rates of EIBF and EBF observed in the Pakistan site in the current study have also been noted in prior investigations [23,24]. It is interesting to note that the Pakistan site differs from others within the Global Network, in that women face many additional risk factors that have been shown, in previous studies, to interfere with EIBF. These include: higher rates of women who have no formal education (83%); women with higher parity (47% parity of 2 or more); later initiation of antenatal care in the 3rd trimester (44%); higher percentage of babies who required resuscitation (5%); and the lowest rate of babies placed on the mother’s chest after delivery (7%) [23-26].

Our study also confirmed several factors generally associated with lack of EIBF such as nulliparity, delivery by caesarean section, the neonate not being put on the mother’s chest after delivery, multiple births, male gender (Africa and Latin America), low birth weight, and if the neonate was resuscitated. Our study supported previous research that delivery by caesarean section is a consistent barrier to EIBF, even in the absence of any neonatal condition that interferes with early initiation of breastfeeding [16,18,27,28]. This is significant, as it delineates a major interventional target by which to improve EIBF in resource-limited settings [29], especially given the recent increase in institutional deliveries and caesarean section rates, particularly in India. There is also a need to reinforce essential newborn care training and education among health workers and families, with emphasis on immediate skin to skin contact after delivery and initiation of breastfeeding within the first hour, especially focusing on low birth weight and premature babies [30-32].

It is unclear why male babies were less likely to have EIBF, but as described elsewhere, there may be cultural beliefs surrounding the birth of males that discourage immediate breastfeeding [23,33-35]. Additionally, in some regions, such as Guatemala, cultural factors such as those related to the belief that colostrum is “dirty” can serve as barriers to EIBF. The role of nulliparity in lack of EIBF may be related to some interplay between maternal age, lack of knowledge, and cultural beliefs, but also provides a group that can be targeted for interventions to improve EIBF rates.

Our study also highlights the importance of EIBF to increase early rates of EBF, at least through day 42 of life. Edmond et al demonstrated that EIBF has the potential to save 22% of neonatal deaths and 16% of all infant deaths [8]. Lack of EIBF may, in particular, be related to an increased risk of mortality due to infection [5,8,36,37]. In our study, lower birth weight is a risk factor for both lack of EIBF and has also been associated with mortality risk. With increasing survival of lower birth weight babies in resource limited settings, interventions to improve EIBF rates should be considered. Our results also highlight the fact that sicker and/or smaller babies are more likely to have feeding problems overall, including inability to initiate early breastfeeding, than their heavier and/or healthier counterparts. And yet, the relationship among birth weight and infant illness is not straightforward; exposure to breast milk may be even more crucial for reduction of morbidity and mortality outcomes, including infection, among the most vulnerable newborns [38]. These complex, multi-factorial, bi-directional associations among infant birth weight, gestational age, delivery complications, maternal characteristics, breastfeeding, and morbidity and mortality outcomes should be further investigated.

The factors associated with lack of EBF were less consistent across the regions than the factors associated with failure to achieve EIBF. The only factor that was significant in all regions for EBF was multiple gestation. However, many of the other factors examined had a significant relationship in sites in one or more regions and points to the necessity to understand this issue in the local context. For example, in Guatemala, several factors including low education, being delivered by a TBA, and starting prenatal care late were protective against failure to achieve EBF at 42 days. Research to understand regional differences in EBF is therefore important.

There are several plausible explanations why our EIBF and EBF rates are higher than in other studies. First, sites in the current study have been part of the Global Network for a number of years [39-57]. These sites have participated in a variety of cluster-based randomized trials to improve maternal and neonatal health including: training of community-based health providers in essential newborn care [46,47]; Emergency Obstetric and Newborn Care [48,49]; Helping Babies Breathe neonatal resuscitation training [50-52]; complementary feeding [53-55] and antenatal corticosteroids [56]. In addition to the MNHR itself, several site-specific efforts have focused on improving case-finding and reporting for a variety of maternal and newborn outcomes [47,58] as well as improved description and classification of facility-based and lay health services [59]. As a result of exposure to these maternal and newborn health care initiatives, it is likely that there is heightened awareness of women, health workers, community opinion leaders, and family stakeholders in these settings about the importance of EIBF and EBF for 6 months.

Second, since data on EIBF is collected shortly after birth, and assessment of EBF occurs on day 42 post-partum, this likely reduces the risk of maternal recall bias, which may have impacted results from cross-sectional DHS surveys and other similar studies. We believe that our data are representative of our communities because the MNHR has high rates of consent, low rates of loss-to-follow up, and well defined variables such as antenatal, delivery, newborn/infant morbidity and mortality, and maternal outcomes.

There are several important strengths of our study. First, we prospectively determined rates of EIBF and EBF at 42 days in a large cohort of women and their babies at 7 sites in 6 low and middle income countries. Second, we prospectively collected data on barriers to EIBF and EBF in a standardized manner, by trained health workers, over a four-year period. Limitations of our study include our reliance on maternal recall about precisely when they initiated breast feeding, although this information is collected within hours or days of birth, not at variable times over months and years. Our rates of EBF at 42 days are based on maternal report using a 24 hour recall method; we did not confirm these maternal reports via observations. In addition, we did not differentiate between “predominant” and “exclusively” breastfed—it is possible that some women may have mistakenly reported that their baby was exclusively breastfed when they were predominantly breastfed. Finally, rates of EIBF and EBF may have been overestimated, particularly if mothers provided a desirable response to the breastfeeding questions because they were familiar the data collectors, and/or had a desire to provide socially appropriate responses [60,61].

A recent systematic review noted the paucity of high-quality data for the “understanding of the independent or combined effects of early initiation and breastfeeding patterns” [36]. We believe that our large, prospective, population-based study of live born neonates at seven sites in Africa, Asia, and Latin America adds to the global evidence-base about risk factors and outcomes of lack of EIBF and EBF. Our study provides an evidence base for specific barriers, within particular global settings, that should be targeted by interventions to improve rates of EIBF and EBF.

## List of abbreviations used

EIBF: Early initiation of breastfeeding; EBF: Exclusive breastfeeding; GN: Global Network; MNHR: Maternal Newborn Health Registry; WHO: World Health Organization.

## Competing interests

The authors’ declare they have no competing interests.

## Authors’ contributions

AP, PLH, and SB conceived of and designed the study, and developed the initial data collection tools specific to the breastfeeding project; AP wrote the first draft of the manuscript, which PLH, SB, RLG and EMM subsequently revised. JLM conducted statistical analyses with DDW and EMM. All the authors participated in the creation and maintenance of the MNHR. All authors read, revised, and approved of the final manuscript.

## Peer review

Reviewer reports for this article can be found in Additional file 1.

## Supplementary Material

Additional file 1 Click here for file

## Declarations

This article has been published as part of *Reproductive Health* Volume 12 Supplement 2, 2015: Research reports from the NICHD Global Network for Women’s and Children’s Health Research Maternal and Newborn Health Registry. The full contents of the supplement are available online at http://www.reproductive-health-journal.com/supplements/12/S2. Publication of this supplement was supported by grants from the Eunice Kennedy Shriver National Institute of Child Health and Human Development to RTI International.
