# Supplementary material for: Rates and determinants of early initiation of breastfeeding and exclusive breast feeding at 42 days postnatal in six low and middle-income countries: A prospective cohort study
Source: Reprod Health. 2015 Jun 8;12(Suppl 2):S10. doi: 10.1186/1742-4755-12-S2-S10 (PMC4464210; doi:10.1186/1742-4755-12-S2-S10)
Supplement: Additional file 1 [file 1742-4755-12-S2-S10-S1.pdf]

Referee's comments to the authors– this sheet WILL be seen by the author(s) and published with the article

Title	Lack of early initiation of breast feeding is associated with late newborn and early infant death among rural populations in 7 low and middle income countries: A prospective cohort study
Author(s)	Archana Patel, Sherri Bucher, Yamini Pusedekar, Fabian Esamai, Nancy F Krebs, Shivaprasad S Goudar, Elwyn Chomba, Ana Garces, Omrana Pasha, Sarah Saleem, Bhala Kodkany, Edward A Liechty, Richard J Derman, Waldemar A Carlo, K Michael Hambidge, Robert L Goldenberg, Fernando Althabe, Mabel Berrueta, Janet L Moore, Elizabeth M McClure, Marion Koso-Thomas, Patricia L Hibberd
Referee's name	Jeanne McDermott

When assessing the work, please consider the following points, where applicable:

1. Is the question posed by the authors new and well defined?
2. Are the methods appropriate and well described, and are sufficient details provided to replicate the work?
3. Are the data sound and well controlled?
4. Does the manuscript adhere to the relevant standards for reporting and data deposition?
5. Are the discussion and conclusions well balanced and adequately supported by the data?
6. Do the title and abstract accurately convey what has been found?
7. Is the writing acceptable?

Please make your report as constructive and detailed as possible in your comments so that authors have the opportunity to overcome any serious deficiencies that you find and please also divide your comments into the following categories:

- Major Compulsory Revisions (which the author must respond to before a decision on publication can be reached)
- Minor Essential Revisions (such as missing labels on figures, or the wrong use of a term, which the author can be trusted to correct)
- Discretionary Revisions (which are recommendations for improvement but which the author can choose to ignore)

Where possible please supply references to substantiate your comments.

When referring to the manuscript please provide specific page and paragraph citations where appropriate.

General comments: Although the use of the MNHR provides a very robust data source, I do not see that this paper adds any new information to better understanding either the barriers to early initiation of breastfeeding (EIBF) or its unique contribution to late neonatal mortality or exclusive breastfeeding at 42 days. As noted in the discussion, the results of their analyses confirm the risk factors identified in other older studies. Unfortunately and disappointingly, their robust data source does not seem to include additional data that would elucidate a better understand of these already know risk factors.

Major compulsory revisions:

- The authors do not clearly define how they selected the samples of mothers and/or babies used for each analysis. Since the sample sizes differed by up to 24,000 (Table 1 and multivariate model in Table 2), a schematic would be useful to reflect missing data, lost to follow-up or deaths among mothers and babies. This is made even more confusing as the authors in some instances do not clearly differentiate mothers from babies used in analyses in the narrative (example, pg. 7, first paragraph under Results – "257,225 "live born babies when this seems to reflect number of women). The term "births" is used in the Tables 1 and 2 without any definition of the term to reflect if includes multiple birth babies. Since the registry covered four years, it is possible that individual women had two births included in the registry, but this is not addressed.
- Lack of consideration of "risk factors" that might be associated with not initiating breastfeeding within the defined "one hour of birth," but might not prevent initiation within 2 hours of birth, such as C/section, assisted delivery, bag and mouth resuscitation, very low birth weight (<1500gms), or multiple birth. It would have been useful to see if any "risk factors" fell out when a more flexible, and

Continued:

possibly more realistic, definition for EIBF is used.

- Lack of inclusion of maternal complications that would be expected to prevent initiation of breastfeeding within one hour of birth, such as postpartum hemorrhage, obstructed labor, prolonged labor, pre- eclampsia/eclampsia, etc.
- In trying to reduce barriers, it is useful to differentiate those which can be addressed from those which are more complicated or impossible to remove.
- No description of how they decided which risk factors to include in the logistic regression models. It seemed that they just included all the risk factors under consideration, without any consideration if they were associated with outcome of interest in a univariate analysis. Inclusion of the univariate risk ratios, 95% confidence intervals and P-values with the multivariate model results in Tables 2, 3, and 5 would allow the reader to assess the level of confounding.
- The sample sizes at each site and the wide variations among the sites in maternal demographic characteristic, delivery practices, and EIBF would seem to allow some site-specific analyses justify including site as a “risk factor” in univariate analyses and possible as a variables in modeling. Additionally, the authors choose not to take advantage of fairly large sample sizes at each site to allow some site-specific analyses or, at least to include sites as variable in univariate and multivariate analyses to look for the possibility of additional unknown factors specific to a site that are affecting the outcomes of interest and might merit further exploration.
- Exclusive Breastfeeding sub-study- Very little description of the purpose of this sub-study, results and implications. While there is a much more robust set of pregnancy and delivery practices included (Table 4), the authors do not explain how or why they were chosen. It seem to be just an add-on to this paper to have more “results.”

Referee's comments to the authors– this sheet WILL be seen by the author(s) and published with the article

Title	Lack of early initiation of breast feeding is associated with late newborn and early infant death among rural populations in 7 low and middle income countries: A prospective cohort study
Author(s)	Archana Patel, Sherri Bucher, Yamini Pusedekar, Fabian Esamai, Nancy F Krebs, Shivaprasad S Goudar, Elwyn Chomba, Ana Garces, Omrana Pasha, Sarah Saleem, Bhala Kodkany, Edward A Liechty, Richard J Derman, Waldemar A Carlo, K Michael Hambidge, Robert L Goldenberg, Fernando Althabe, Mabel Berrueta, Janet L Moore, Elizabeth M McClure, Marion Koso-Thomas, Patricia L Hibberd
Referee's name	Yvonne Vaucher

When assessing the work, please consider the following points, where applicable:

1. Is the question posed by the authors new and well defined? Yes
2. Are the methods appropriate and well described, and are sufficient details provided to replicate the work? Yes, See below
3. Are the data sound and well controlled? Yes
4. Does the manuscript adhere to the relevant standards for reporting and data deposition? Yes
5. Are the discussion and conclusions well balanced and adequately supported by the data? Yes, See below
6. Do the title and abstract accurately convey what has been found? Yes
7. Is the writing acceptable? Yes

Please make your report as constructive and detailed as possible in your comments so that authors have the opportunity to overcome any serious deficiencies that you find and please also divide your comments into the following categories:

- Major Compulsory Revisions (which the author must respond to before a decision on publication can be reached)
- Minor Essential Revisions (such as missing labels on figures, or the wrong use of a term, which the author can be trusted to correct)
- Discretionary Revisions (which are recommendations for improvement but which the author can choose to ignore)

Where possible please supply references to substantiate your comments.

When referring to the manuscript please provide specific page and paragraph citations where appropriate.

General comments: The primary outcome of this study was to determine the rate of early initiation of breastfeeding (EIBF). Secondary outcomes included determination of factors associated with failure of EIBF and the relationship between EIBF and mortality in the first 6 weeks of life in 7 large, multinational, rural cohorts who were participants in the NICHD Global Network Maternal and Newborn Health Registry. The results of the study support the role of EIBF in reducing neonatal and early infant mortality.

The strengths of the study include prospective, comprehensive, standardized, demographic and outcome data collection proximate to the events studied over a four year period, large sample size and worldwide representation from countries in Latin America, Africa and S. Asia.

Although the entire sample (N=257,225) was used to determine the primary outcome, a much smaller subsample (N=31,608) was available for analysis of the secondary outcomes. However, subjects were unequally distributed from different regions with S. Asia contributing the majority of subjects (>60%) in both the large and smaller samples and in addition, Pakistan is a clear outlier for the primary outcome raising the question of whether the data should be presented as site specific rather than combined. If the data are combined, this inequality should be addressed statistically.

(continue on the next sheet)

Continued: **The authors should describe in the Methods section the type of longstanding participation of these cohorts in the Global Network as well as how the larger and small sub-sample differ (i.e. the sub-sample surveyed included only those women who received ANC and EBF recommendations. These descriptions are in the discussion but are important for the reader to understand before the results are presented. It would be helpful if the authors explored potential explanations for the higher rates of EIBF compared to previous surveys and discuss the generalizability of their findings with respect to the rate of EIBF and the rates of EBF at 6 weeks.**

Did the analysis for risk factors for mortality include country site and male gender?

Figures: Per the paper Pakistan EIBF should be 24% on Figure 1. Please use the same Y axis scale for both Figures.

Supplement Editor comments

There are great concerns regarding your manuscript including its originality and some of the associations and analyses. We wonder if you will be able to perform many major changes. If so, we will consider the possibility to reconsider your manuscript and to look for re-revision.

In the view of the editors the focus of this manuscript should be the description of early initiation of breastfeeding and exclusive breastfeeding at 42 days at population level in communities of various countries.
We do not see the value of analyzing the information of the 6 countries together looking for the association with risk factors.
Also we consider that analysis of the association of EIBF with neonatal mortality should be removed. An interesting analysis would be to look for factors that could explain the differences in the rates of EIBF and EBF among the countries.
In sum our advice is to look for a descriptive study focusing in the differences in the rates among the countries.
